# Editorial: Food of the future: Algae and aquaculture

**DOI:** 10.3389/fnut.2023.1177886

**Published:** 2023-03-21

**Authors:** Qian Lu, Slađana Rakita, Carmen Navarro-Guillén

**Affiliations:** ^1^School of Grain Science and Technology, Jiangsu University of Science and Technology, Zhenjiang, China; ^2^Institute of Food Technology, University of Novi Sad, Novi Sad, Serbia; ^3^Departamento de Biología Marina y Acuicultura, Instituto de Ciencias Marinas de Andalucía (ICMAN-CSIC), Puerto Real, Spain

**Keywords:** algae, aquaculture, sustainability, nutrient bioavailability, human health

Food and feed shortage are serious problems challenging the sustainable development of human society. Besides, food-related health is becoming an emerging topic attracting researchers' attentions. In recent years, more and more studies are exploiting new food resources from algae and aquaculture and also exploring novel technologies for the improvement of nutritional values of food. The Research Topic entitled “*Food of the Future: Algae and Aquaculture*” emphasizes on the advanced technologies for the sustainable development of aquaculture and exploitation of algae as functional food. Articles of this Research Topic cover the aspects of algae-based aquatic food, genomic analysis of algae and fish, and algae-based functional diet.

The study of Chen et al. explored the feasibility of using *Chlorella pyrenoidosa* as an alternative ingredient in aquaculture. The experimental results demonstrated that the microalgae contain some key nutrients, such as essential amino acids and unsaturated fatty acids, as well as bioactive compounds, which are essential for the growth and immune responses of aquatic organisms. The high protein content (>52%) in *Chlorella pyrenoidosa* is regarded as a great advantage of using algae an ingredient in aquafeeds. It is noteworthy that this study firstly discovered the presence of anti-nutritional factors (ANFs) in algae and compared algae and soybean meal in relation to ANF content. The analysis of molecular characterization of proteins in algae, fish meal, and soybean meal showed that high-molecular-weight proteins in algae may be a factor limiting the application of algal biomass as fish diet, and suggested the adoption of pretreatment to lower the molecular weights of proteins and improve their digestion. Based on the work of Chen et al. both advantage and disadvantage of growing algae for aquafeeds are discovered, providing an objective opinion on the exploitation of algae-based fish diets. Accordingly, appropriate technologies can be employed to eliminate the limiting factors in algae for aquaculture practices.

In the study of Manoppo et al. *Caulerpa lentillifera*, a species of green algae, was provided to rat fed with a cholesterol- and fat-enriched diets (CFED) to ameliorate obesity-related metabolic disorders. It was discovered that supplementation of *Caulerpa lentillifera* effectively reduced blood glucose level and total cholesterol level in CFED rats. In addition, the increments of peroxisome proliferator-activated receptor γ coactivator-1α and liver superoxide dismutase were observed in CFED rats supplemented with *Caulerpa lentillifera*, confirming the contribution of algae to the improvement of antioxidant capacity in CFED rats. As a consequence, cellular energy metabolism of rat is impacted owing to the changes of antioxidant properties in the internal environment of rat. Manoppo et al. also pointed out that anti-hyperglycemic activity of *Caulerpa lentillifera* is partly attributed to a variety of bioactive molecules, such as sulfated polysaccharides and monosaccharides. Finally, a dose of 150 mg/kg of body weight was identified as an effective way to ameliorate metabolic problems caused by CFED in rats. Potentially, the dose achieved from these preclinical trials could be a reference for future clinical trials in humans.

Radwan et al. studied the dual effect of dietary supplements of *Grateloupia acuminata* and *Grateloupia doryphore* nanoparticles (GNS) at different concentrations with bionanocomposite cellulose acetate membranes (CA/bio-AgNps) on growth performance and health status of *Oreochromis niloticus*, a species of Nile tilapia native to the Levante area and the northern half of Africa. The results showed that supplementing GNS diets improved the growth rate, weight gain, carcass weight, and crude protein content of tilapia. In addition, survival ratios of tilapia supplemented with GNS diets (T5, T4, T3, and T2) in the *Aeromonas hydrophila* challenge test were much higher than that of tilapia in the control group. Antioxidative, stress-related, and proinflammatory-related genes were up-regulated with the addition of GNS in fish diets. It was also discovered that the aquaculture water after filtering through CA/bio-AgNps contained much lower concentrations of some chemical and bacteriological parameters. Therefore, dietary supplements of GNS with CA/bio-AgNps could positively impact growth performance, immune response, and health status of Nile tilapia.

The first genome-wide association study was performed by Luo et al. to demonstrate the markers and candidate genes associated with seven growth traits of the rice flower carp, which is an important economic fish species of integrated rice-fish culture in China. In addition, it was discovered that B6_4352672 and A8_4978825 were significantly associated with more than five growth traits of the rice flower carp. Among 411,913 loci obtained from the reduced-representation genome, 90 loci were significantly associated with the growth traits of carp. Particularly, *ccdc92, nbeal1, prkn, adipor2, trim25, tlr13, usp49, otud5, elk1*, and *nfic* were associated to the metabolism, immunity, osteogenic differentiation, and visual function. Luo et al. emphasized that the supply of rice flower carp can be increased to match increasing market demand by employing growth-trait associated markers for the improvement of fish growth ratio. In general, Luo et al. not only revealed the genetic mechanisms related to rice flower carp growth, but also provided a potential way to increase rice flower carp production for market.

A genomic selection (GS) model was trained and a mixed-ploidy additive relationship matrix was employed by Huang et al. to predict the breeding values of the haploid gametophytes of *Saccharina latissima*, which is economically and ecologically important in the North Atlantic Oceans and the eastern North Pacific. It should be mentioned that this empirical research conducted by Huang et al. is the first to report the applicability of genomic prediction of haploid breeding values. The results of this work can be applied to shorten the time per breeding cycle of *Saccharina latissima* by predicting the breeding values of non-phenotyped individuals. In addition to *Saccharina latissima*, the developed GS model developed by Huang et al. may be applicable for some other bi- and tri phasic algae, such as *Gracilaria* sp. and *Asparagopsis* sp.

In summary, articles of this Research Topic provide recent progresses and insights on advanced technologies in the industries of algae and aquaculture. Some of the results obtained from these studies are expected to solve food shortage globally and improve the health status of humans in modern society.

## Author contributions

QL contributed to manuscript writing. CN-G and SR contributed to manuscript revision. All authors contributed to the article and approved the submitted version.

